# Supplementary material for: Interlaboratory Validation of a DNA Metabarcoding Assay for Mammalian and Poultry Species to Detect Food Adulteration
Source: Foods. 2022 Apr 12;11(8):1108. doi: 10.3390/foods11081108 (PMC9027865; doi:10.3390/foods11081108)
Supplement: Supplementary file 1 [file foods-11-01108-s001.zip › foods-1646732-supplementary.pdf]

foods

Supplementary Table S1. Sequences included into the reference database.

Accession number	Scientific name of the species
NC_000845	*Sus scrofa*
NC_006853	*Bos taurus*
NC_006295	*Bubalus bubalis*
NC_012346	*Bison bison*
NC_014044	*Bison bonasus*
NC_001640	*Equus caballus*
KT_368730	*Equus caballus*
NC_001788	*Equus asinus*
NC_018781	*Equus quagga*
NC_007704	*Cervus elaphus*
KT_290948	*Cervus elaphus hippelaphus*
NC_013834	*Cervus nippon*
NC_020684	*Capreolus capreolus*
NC_001941	*Ovis aries*
KR_866125	*Capra hircus*
NC_020633	*Rupicapra rupicapra*
NC_020623	*Capra ibex*
NC_007703	*Rangifer tarandus*
NC_020677	*Alces alces*
NC_004028	*Lepus europaeus*
NC_001913	*Oryctolagus cuniculus*
NC_020678	*Antidorcas marsupialis*
NC_001794	*Macropus robustus*
NC_027424	*Macropus giganteus*
KY_996501	*Macropus rufus*
NC_028625	*Castor fiber*
NC_033912	*Castor canadensis*
NC_002008	*Canis lupus familiaris*
NC_008092	*Canis lupus*
NC_001700	*Felis catus*
NC_008434	*Vulpes vulpes*
NC_009629	*Camelus ferus*
NC_012102	*Lama glama*
NC_012374	*Rattus rattus*
NC_001665	*Rattus norvegicus*
NC_005089	*Mus musculus*
NC_025952	*Mus spretus*
NC_008142	*Crocodylus niloticus*
NC_001323	*Gallus gallus*
NC_010195	*Meleagris gallopavo*
NC_010965	*Cairina moschata*
NC_011196	*Anser anser*
NC_002785	*Struthio camelus*
NC_015526	*Phasianus colchicus*
NC_009684	*Anas platyrhynchos*
NC_006382	*Numida meleagris*
NC_013978	*Columba livia*
NC_020585	*Alectoris chukar*
NC_003408	*Coturnix japonica*
NC_039843	*Perdix perdix*
NC_000884	*Cavia porcellus*

Supplementary Table S2. Total number of reads after pipeline (n=14, samples 1 - 7, two subsamples each). min: minimal value, max: maximal value, mean: arithmetic mean, RSD: relative standard deviation.

Laboratory	Sequencing platform	AGES database				NCBI database			
		min	max	mean	RSD (%)	min	max	mean	RSD (%)
01	MiSeq	112867	332337	160951	34.2	112929	332478	161433	34.0
02	MiSeq	30685	266885	206082	28.5	30935	267014	206609	28.5
03	MiSeq	197147	411034	304147	21.1	197715	411146	304825	21.1
04	MiSeq	201332	250906	224860	6.4	201383	251768	225412	6.5
06	MiSeq	201936	377693	239369	17.8	202237	378268	240033	17.7
07	iSeq	81300	347382	194085	33.6	81569	348324	194550	33.6
08	MiSeq	159322	239781	196462	12.3	160460	240628	196997	12.3
09	Ion GeneStudio S5	301779	530818	435239	14.9	303112	530996	436858	14.8
10	Ion GeneStudio S5	291375	614742	469803	20.3	291860	618393	471319	20.3
11	Ion GeneStudio S5	299993	408425	367537	8.3	301224	408556	368651	8.3
12	Ion GeneStudio S5	434327	611296	513356	11.8	436685	612995	515019	11.8
13	iSeq	116993	213126	157863	16.7	117623	213195	158200	16.6
14	MiSeq	138357	205768	161638	11.1	138787	206287	162034	11.1
15	MiSeq	115016	209082	170278	16.9	115498	209699	170769	16.9
20	iSeq	89306	144455	120823	14.6	89567	144774	121113	14.5

Supplementary Table S3. Recovery (%) (total number of reads after pipeline related to the number of raw reads before analysis pipeline). (n=14, samples 1 - 7, two subsamples each). min: minimal value, max: maximal value, mean: arithmetic mean, RSD: relative standard deviation.

Laboratory	Sequencing platform	AGES database				NCBI database			
		min	max	mean	RSD (%)	min	max	mean	RSD (%)
01	MiSeq	72.6	87.9	82.5	5.4	72.9	88.1	82.7	5.5
02	MiSeq	71.0	93.3	89.7	6.4	71.6	93.6	90.0	6.3
03	MiSeq	74.7	90.8	84.1	6.1	74.9	91.3	84.3	6.2
04	MiSeq	91.4	95.2	92.9	1.3	91.5	95.2	93.2	1.3
06	MiSeq	90.5	95.5	93.0	1.9	90.8	95.5	93.2	1.8
07	iSeq	82.1	92.7	87.1	3.4	82.6	92.7	87.3	3.2
08	MiSeq	86.4	94.0	91.0	2.6	86.6	94.1	91.3	2.6
09	Ion Gene Studio S5	91.4	96.9	94.6	1.8	91.6	96.9	95.0	1.8
10	Ion Gene Studio S5	82.5	97.0	93.2	4.6	82.6	97.1	93.5	4.7
11	Ion Gene Studio S5	94.6	97.7	96.3	1.0	95.2	97.7	96.6	0.8
12	Ion Gene Studio S5	94.1	97.4	95.9	1.0	94.7	97.4	96.2	0.9
13	iSeq	78.6	96.1	88.7	6.8	79.0	96.1	88.9	0.1
14	MiSeq	92.2	96.7	94.2	1.5	92.5	96.7	94.4	1.4
15	MiSeq	81.8	90.5	86.4	3.2	82.2	90.6	86.6	3.1
20	iSeq	67.5	77.9	72.7	5.3	67.7	78.1	72.9	5.2
